# Idiopathic Symptoms Resolved by Pharmacogenomics-Enriched Comprehensive Medication Management: A Case Report

**DOI:** 10.7759/cureus.21834

**Published:** 2022-02-02

**Authors:** April Prather, Aissa Aifaoui, Jeffrey A Shaman

**Affiliations:** 1 Clinical Pharmacy, Know Your Rx Coalition, Lexington, USA; 2 Clinical Sciences, Coriell Life Sciences, Philadelphia, USA

**Keywords:** polypharmacy, clinical decision support system, drug metabolism, cytochrome p-450, adverse drug reactions, pharmacogenomics, personalized medicine, healthy aging, aging

## Abstract

Clinical manifestations of biological aging can be remarkably similar to the side effects of frequently used medications. Fatigue, muscle pain, and confusion are common and often not shared as part of proper geriatric patient history. When patients report them, a root cause is usually confounding. These symptoms not only negatively impact health and wellness outcomes, patient quality of life, and increase costs to the healthcare system, but also may be a limitation on provider best practices. The patient, a 71-year-old female of European descent, enrolled in pharmacogenomics-enriched comprehensive medication management (PGx+CMM) program through her retirement benefit. At the time of testing, she was approximately 18 months post cerebrovascular accident and was being observed by her primary care provider for common chronic conditions. Of interest, she had been manifesting unreported clinical symptoms of fatigue, hypotension, and myalgia. Addressing these patient concerns and specifically focusing on an individual’s goals, fears, and basic needs, rather than concentrating merely on the absence of disease, is the crux of personalized medicine and programs that address the notion of healthy aging. The patient’s therapeutic regimen was adjusted based on PGx+CMM pharmacist review, use of a clinical decision support system (CDSS), and communication of recommendations to the prescribing physician. The patient saw rapid improvements in symptoms, suggesting they were caused by medication side effects. Her blood pressure and cholesterol levels remained controlled while noticeable side effects were eliminated. This case study demonstrates the positive impacts of personalized medicine and shows how pharmacists can be empowered with a CDSS to positively impact healthcare.

## Introduction

Biological aging is commonly referenced as an inherent decline in physical function over time. It is a complex process characterized by a progressive decline of numerous physiological, cellular, and molecular functions [1,2] and symptoms such as hearing loss, fatigue, weight loss, and reduction in energy and visual acuity [3,4]. As the human body naturally changes as we age, pharmacological interventions are routinely used to prevent, treat, or cure diseases that may arise [5]. The coexistence of ischemic heart disease, hypertension, and hyperlipidemia, a common triad of chronic conditions, is prevalent among Medicare beneficiaries over the age of 65 [6-8]. This combination of diseases often results in a patient being chronically prescribed medications such as beta-blockers for blood pressure control, statins for hyperlipidemia, and anticoagulants to prevent blood clot formation [9]. Undesirable side effects of these commonly prescribed medications include fatigue, depression, insomnia, loss of appetite, diarrhea, easy bruising, bleeding, and myalgia [10]. These side effects can negatively impact a patient’s quality of life and can present an obstacle to healthy aging, especially when added to the symptoms of the chronic conditions being treated.

Healthy aging, as defined by the World Health Organization (WHO), the Centers for Disease Control and Prevention (CDC), and others, includes the process of developing and maintaining the optimal physical, mental, social, and functional ability that enables wellbeing in older age [11,12]. Advocates for healthy aging aim to shift the definition of “health” from the absence of disease towards the cultivation and preservation of functional abilities throughout life. For example, in the healthy aging concept, quality of life can be measured by the ability of an individual to have control of the basic and valued needs - the presence of intact mental and physical functions in order to live independently [13,14]. These innate capabilities can be significantly diminished in the older population by factors such as biological aging, the presence of multiple diseases, and imperfect pharmacotherapy [15]. Various approaches have been developed to address these factors, including outcomes-based payment models (e.g., Medicare’s Quality Payment Program), integrated care models (e.g., Geriatric Patient-Aligned Care Teams, GeriPACT, in the Veterans' Health Administration), and comprehensive in-home care models (e.g., Geriatric Resources for Assessment and Care of Elders, GRACE, Team Care). Regardless of the approach, the central goal of geriatric medicine is to help older adults experience healthy aging.

Providers, however, often face a number of challenges when diagnosing persons 65 years and older, including distinguishing between physiologically accepted aspects of biological aging and those which are preventable or treatable. To assist with these issues, providers now have access to additional diagnostic tools - namely pharmacogenomics, clinical decision support systems (CDSSs), and collaborative agreements with specialists such as pharmacists - to facilitate the practice of personalized medicine.

Personalized medicine incorporates comprehensive, multidimensional clinical decision-making to maximize the outcomes an individual patient values most and to minimize factors the patient fears the most [16,17]. Pharmacogenomics (PGx), the application of how a person’s genetic biomarkers impact their response to medications, has proven instrumental in empowering personalized medicine and more specifically in improving the health of patients suffering from depression, cardiovascular disease, polypharmacy, and other conditions [18]. PGx is clinically beneficial because variations in the genes that encode proteins that transport drugs across membranes and the genes that metabolize medications can impact the clinical outcome of specific therapeutic agents [19].

The list of clinically impactful variations and therapeutic responses to specific genetic biomarkers continues to expand [20,21] and includes medications in various therapeutic classes with clinically actionable information [22,23]. As an example, the *5 allele (Val174Ala, rs4149056) in the hepatic drug transporter SLCO1B1 is associated with simvastatin-induced side effects [24,25]. This variant leads to higher systemic simvastatin concentrations by interfering with the proper plasma membrane localization of the transporter [26]. Variants in the *CYP2D6* gene alter the ability and rate of the CYP2D6 enzyme to metabolize and eliminate medications including metoprolol, a β-blocker. Patients with a decreased rate of metoprolol metabolism by CYP2D6 will have higher plasma concentrations of metoprolol and may be at an increased risk of side effects [27-29] including a decreased cardioselectivity [30]. Experts suggest choosing a different statin and adjusting the metoprolol dose in response to variants detected in genetic tests in order to avoid potentially dangerous side effects and efficacy concerns.

Assaying these and other clinically relevant genetic biomarkers involves an inexpensive, non-invasive laboratory test, the results of which complement complete medication safety reviews to identify risk and efficacy and to guide prescribing. A comprehensive medication safety review includes evaluating the risks of decreased efficacy or increased toxicity due to drug-drug interactions, contraindications, and lifestyle factors.

## Case presentation

A 71-year-old female of European descent was approximately 18 months post cerebrovascular accident and was being observed by her primary care provider (PCP) for chronic conditions including hypertension, hyperlipidemia, atrial fibrillation, and gastroesophageal reflux disorder. The patient reported being concerned about the sudden increase in medications prescribed after her stroke and wanted to be sure that her medications were safe and appropriate for her as an individual. Concurrently, she was invited to participate in pharmacogenomics-enriched comprehensive medication management (CMM) program through her retirement benefit. She discussed the program with her PCP who encouraged her to participate.

The patient submitted a saliva sample via a DNA testing kit (Oragene•Dx® OGD-510, DNA Genotek, Ontario, Canada) and consent was obtained. This study was approved by the Biomedical Research Alliance of New York Institutional Review Board (BRANY; approval #20-15-600-753). Her DNA was subsequently analyzed by a CLIA- and CAP-licensed laboratory (Quantigen, Fishers, IN), and the results were made available to the pharmacist for review in GeneDose LIVE™ (Coriell Life Sciences, Inc., Philadelphia, PA, version 2.1), a CDSS. Pharmacogenomic results for 14 of the 16 genes assayed were clinically unexceptional considering her medication regimen. Atypical, relevant results were reported for *CYP2D6* *1/*6 and *SLCO1B1* *1/*5. The clinical pharmacist contacted her by telephone to confirm her complete medication regimen, document her relevant lifestyle factors (e.g., alcohol, tobacco use), and determine any potential medication-related issues that she may have been experiencing.

**Figure 1:**
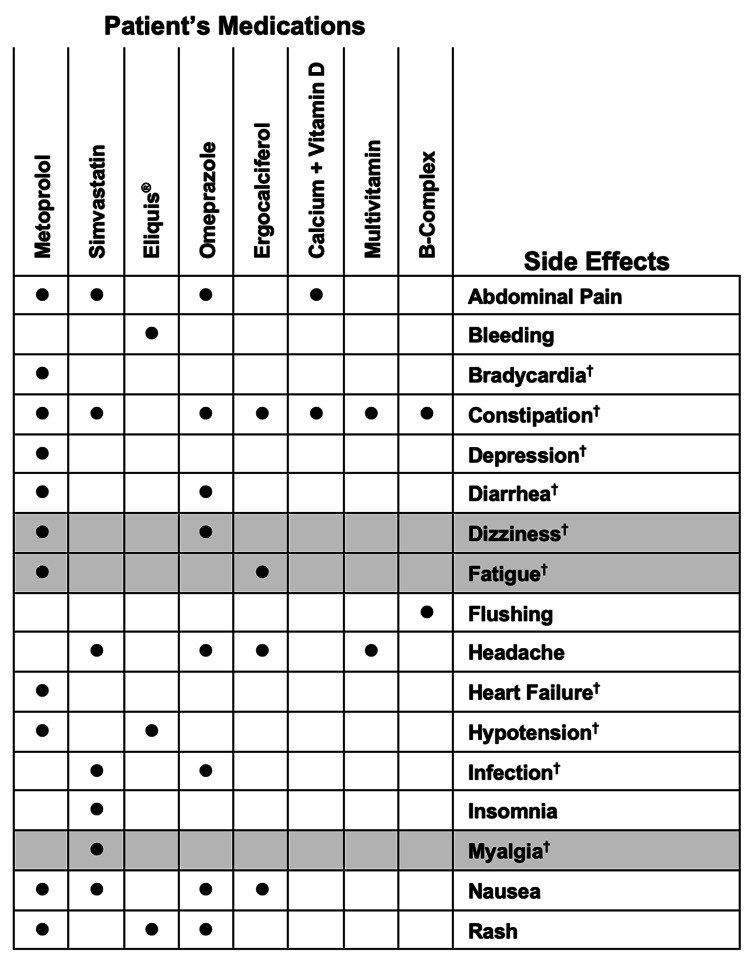
Patient medication list and commonly reported side effects The patient reported taking these medications as prescribed by her primary care physician. These medications have common side effects ($\cdot$). Symptoms known to be associated with biological aging are noted (†). The patient specifically reported symptoms of dizziness, fatigue, and myalgia (highlighted in grey). The intersection of likely causes - medication and biological aging - with a symptom illustrates the challenges to identifying the source of the patient’s idiopathic symptoms.

At the time of medication review she reported that her medication regimen contained metoprolol succinate extended-release, 25mg once daily; simvastatin, 40mg once daily; Eliquis® 5mg, once daily; omeprazole 40mg once daily; ergocalciferol 1.25mg once weekly, calcium carbonate 600mg plus vitamin D 200 IU; a complete multivitamin supplement, and a B-complex multivitamin (Figure 1). Additionally, she reported a history of hypertension, hyperlipidemia, atrial fibrillation, stroke, gastroesophageal reflux disorder, vitamin D deficiency, and osteoporosis. She listed symptoms of fatigue, occasional dizziness when standing, nightly bilateral leg pain or cramping, and general low energy. She described having felt less energetic for some time but expressed that she felt it was expected due to her decreased cardiovascular health and increased age.

Pharmacogenomics, demographics, lifestyle factors, indications, and medication regimens were captured and incorporated in the CDSS for the CMM review. For the pharmacist review, the CDSS displayed the risks across nine potential sources that were associated with each of the patient’s medications.

Specifically, the CDSS identified two warnings from the American Geriatric Society's Beers Criteria® for Potentially Inappropriate Medication Use in Older Adults [31], one US FDA “black box warning,” an anticholinergic burden risk, and two clinically-significant genetic interactions associated with the patient’s medication regimen. The pharmacogenomic report identified her as an intermediate metabolizer of metoprolol by the CYP2D6 enzyme. It also detailed her intermediate (i.e., decreased) liver uptake of simvastatin by SLCO1B1. There were no contraindications or warnings associated with the patient’s diet discovered.

The pharmacist used the CDSS to evaluate alternative medications, in silico, to ultimately assist in addressing the efficacy and side effect concerns that were surfaced. After a telephonic conversation with the patient and a thorough review of the risks, the pharmacist determined that suitable alternatives for metoprolol succinate 25mg and simvastatin 40mg in the context of the medication regimen were bisoprolol 5mg and rosuvastatin 5mg, respectively. Bisoprolol is an alternative β-blocker that is not dependent on CYP2D6 metabolism; patient response to rosuvastatin is not affected by SLCO1B1 polymorphisms. Using the CDSS, the pharmacist subsequently created a medication action plan (MAP) that summarized the proposed regimen changes for the patient’s primary care physician. The MAP highlighted the medications' safety concerns, provided a brief rationale for recommended changes, and included a pre-filled prescription order form for physician convenience. The primary care provider reviewed the MAP and adjusted the patient’s therapeutic regimen based on each of the pharmacist’s recommendations. Specifically, metoprolol succinate, 25mg once daily was modified to bisoprolol, 5mg; simvastatin, 40mg once daily was replaced with rosuvastatin, 5mg.

At 18 months post pharmacist review, the patient reported that she continued taking each of the recommended medications as prescribed. She reported feeling more energetic-she was able to attend her workout class more frequently. She also described being more well-rested and attributed it to the lack of leg pain while sleeping. Her PCP reported that her blood pressure and cholesterol levels remained moderately controlled and that no further adjustments to her medications had been made in the preceding 18 months.

## Discussion

In this case study, we described the positive health outcomes of a patient that enrolled in a pharmacogenomic-enriched comprehensive medication management program offered as a benefit through her state-run retirement system’s health plan. Specifically, she realized a decrease in unwanted symptoms, an increase in self-reported quality of life, and positive interactions with her healthcare providers. The patient originally reported a history of fatigue, dizziness, leg pain, and low energy, each of which is a common symptom among geriatric patients. It is highly probable that medication changes were not previously considered by the patient’s physician because her blood pressure was within the recommended normal range at each visit, demonstrating treatment efficacy. Additionally, the patient acknowledged the failure to report her symptoms to her doctor because she felt these symptoms were “typical for old folks like me.” Confounding the issue was the idiopathic nature of these symptoms-the causes could be biological aging, side effects of medications, or undiagnosed morbidities.

Pharmacogenomics proved to be an important factor in uncovering the cause of these symptoms and ultimately guiding the treatment adjustments that proved successful for this patient. The decreased metabolism of metoprolol and transport of simvastatin were consistent with symptoms stemming from the side effects of medications rather than biological aging. This alone did not exclude biological aging as the cause. However, a positive reduction in side effects was appreciated for >18 months after the medication regimen was adjusted - with no negative impact on therapeutic intent - suggests that medication side effects were the cause of this patient’s symptoms.

Optimizing drug therapy in the older population experiencing polypharmacy and multi-morbidities continues to be a constant challenge as age-related changes in pharmacokinetics and pharmacodynamics significantly increase the risk of adverse outcomes [32]. Furthermore, clinical acceptance and implementation of CMM, and separately pharmacogenomic testing, have been only mildly successful in most countries. The greater challenge has been how to employ the combined insights of pharmacogenomics and CMM, at the population scale, and in the context of the fragmented health delivery models that exist today. Any such application would need to arm healthcare providers with additional tools to accurately identify and resolve issues of inappropriate medication use with speed and accuracy and to deliver those results to be actioned.

The CDSS used in the PGx+CMM) program described in this case study functionally connected three fundamental components-education and enrollment, pharmacogenomic testing, and pharmacist review. Briefly, the patient was first provided with educational materials regarding what pharmacogenomics can and cannot do, an understanding of the risks and benefits, issues of privacy and cost, and a method for enrolling. Next, the patient’s DNA sample was analyzed by the laboratory and the results were added to the CDSS. Finally, a trained pharmacist performed the PGx+CMM review, utilizing the comprehensive CDSS to integrate and communicate medication adjustment suggestions via a concise MAP. The pharmacist was then empowered to communicate the results to the patient’s prescribing physician. The pharmacist was the specialist at the hub of the patient’s care using the CDSS, they were the learned intermediary able to discover, communicate, and impact change.

The incorporation of personalized medicine into this older adult patient’s care plan provides an example of how health systems could improve patient outcomes while supporting the goals of healthy aging. This patient experienced a quality-of-life improvement measured by increased capacity to complete the activities she valued most. As she felt healthier, she was able to increase her physical activity. This could lead to other improvements to her health and wellbeing such as reductions in blood pressure, weight, and cholesterol. The ability to interact freely within her community also stimulates improved mental health as a sense of wellbeing is regained. She was also able to decrease her out-of-pocket costs on prescription medications when the pharmacist identified a lower-cost pharmacy for one of her maintenance medications. This case validated an integrative, PGx+CMM program that can positively impact a patient and their healthcare provider. Further investigation of this program is warranted to understand the impact on a larger population and should include metrics (e.g., economic, clinical, and humanistic outcomes) for the Quadruple Aim in healthcare.

## Conclusions

While biological aging is a natural process of decline, innovative programs may ensure that patients experience healthy aging by focusing on patient-centered values and outcomes. Polypharmacy, multimorbidity, and the resulting increased risk of poor health outcomes are prominent among geriatric patients. Adverse drug reactions can potentially mimic signs of natural aging, further confounding the ability of providers to identify the cause of symptoms. Genetic testing is an integral component of personalized medicine that facilitates the quick, accurate, and appropriate medication selection to decrease side effects and increase therapeutic efficacy. This clinical case has demonstrated that a process that integrates pharmacogenomics with pharmacist review can improve patients’ quality of life and positively contribute to healthy aging.
